# The IWJ turns 20

**DOI:** 10.1111/iwj.14025

**Published:** 2022-11-28

**Authors:** Douglas Queen, Keith Harding

It's been a while since we focused our end of year editorial on the journal itself. COVID kind of swamped the airwaves so to speak. But as the International Wound Journal enters its 20th year in 2023, we decided that it's time to reflect not only on the past year but the entire lifespan of the journal.

When we were developing the idea of a truly international offering in the wound care space, where geographies are at different stages of the evolution of wound care as a specialty, we wanted it to have equitable access for all researchers and readers. Some 20 years on we are proud of what Wiley and the IWJ team have developed and recognise that the IWJ of today remains true to that initial vision. In the past few years, we moved from a subscription‐based model to full open access, being the first wound care focussed journal to do so. This step also remained true to the equitable access vision and was essentially a move based on the evolving publication mechanisms of today.

As the world of research and publication evolves, the IWJ has and will evolve to meet the needs of its authors and readers. Since its inception in 2004 the IWJ has always been at the leading edge of publication trends.

Our impact factor has grown year on year and the IWJ remains one of the top three journals in the wound care field. Citation is only one of the measures of success, albeit an important one. Downloads are another measure of interest and utility. Over the years our downloads have increased steadily but not surprisingly took an exponential jump upon open access. In 2022, we are on track to reach a million downloads. This shows the value of the IWJ in the field of wound care.

Reaching these lofty goals and remaining one of the worlds most valued resources did not happen alone. Yes, the team at Wiley and our IWJ editorial team started and maintained some momentum of success, but it is our contributors and readers that achieved this success. Think of the IWJ team as the engine and the contributors and readers as the fuel. Both are required for successful operation of the vehicle.

So, as we approach our 20th birthday in March 2023, we would like to thank everyone who has ever contributed to the IWJ and everyone who has ever read its contents. We truly appreciate your efforts and support and here is to the next 20 years of the International Wound Journal.

